# Comment on Inácio et al. Nursing Practice Environment in the Armed Forces: Scoping Review. *Nurs. Rep.* 2025, *15*, 394

**DOI:** 10.3390/nursrep16040114

**Published:** 2026-03-31

**Authors:** Giovanni Cangelosi

**Affiliations:** 1 School of Pharmacy, Experimental Medicine and “Stefani Scuri” Public Health Department, University of Camerino, 62032 Camerino, Italy; giovanni01.cangelosi@unicam.it; 2 Scientific Commitment of Italian Coordination of Volunteer Nurses for Health Emergencies Association (CIVES), Via Agostino De Pretis 70, 00184 Rome, Italy; 3 Public Health Research Group of Italian Society of Nephrology Nurses (SIAN), Via Capotesta 1/30, 07026 Olbia, Italy

I have read the article entitled “Nursing Practice Environment in the Armed Forces: Scoping Review” by Inácio et al. published in Nursing Reports, 2025 Nov 7;15(11):394 [1]. I want to congratulate the authors for this successful review article, and I believe this work con-stitutes a very important contribution to the scientific community.

The manuscript presents a comprehensive analysis of the nursing practice environment (NPE) in military contexts, emphasizing the importance of leadership, collaboration, professional development, and adequate resources in determining nurse well-being, retention, and patient safety. The work stands out for its solid methodological rigor [2,3], which confers both scientific and practical validity to the manuscript, making the study valuable not only to the journal’s readers but also to the wider academic community, thus offering a highly reliable contribution in a still underexplored field [1,2,3]. 

This comment reflects on the broader relevance of NPE dynamics for care quality and nurse well-being, emphasizing their potential applicability across different organizational and operational contexts without extending beyond the scope of the original study.

## 1. Nursing Well-Being and Burnout Prevention

As demonstrated in the study of Inácio et al. [1], in military settings, the nursing practice environment is a central dimension in relation to nurse well-being, burnout, and staff retention. Based on this concept, the literature on nurses working in high-complexity and emotionally demanding civilian settings—such as emergency- and urgent-care services and correctional institutions—shows that high emotional workloads and rigid, and at times outdated, organizational structures significantly contribute to the development of burnout and professional stress, with potential negative repercussions on quality of care, patient safety, and continuity of care [4,5,6,7,8]. Taken together, this evidence reinforces the idea that the quality of the work environment represents a cross-cutting and universal determinant of professional well-being, particularly for healthcare professionals such as nurses, who, in military settings, operate under conditions marked by high levels of clinical, organizational, and emotional complexity. These professionals are frequently exposed to high-risk scenarios, strict operational and decision-making constraints, and substantial ethical and moral challenges, all of which may exert an indirect yet significant influence on care outcomes and the overall quality of care delivery [9,10,11,12].

## 2. Nursing Leadership and Care Management in Complex Setting

The study by Inácio et al. [1] highlights adaptive leadership as a key structural factor in the military nursing practice environment. Leaders capable of modulating transformational and directive styles contribute to trust, team cohesion, and patient safety [13]. In military contexts, operational complexity, hierarchical structures, and frequent rotations require nurses to demonstrate strategic and operational flexibility. The capacity to alternate between directive and collaborative approaches in both crisis and routine contexts has been reported to support stress management, conflict reduction, and staff retention [14].

Similar organizational and emotional demands are observed in civilian contexts, such as in the care of migrant patients, where effective management of cross-cultural relationships requires skills in communication, negotiation, and empowerment [6,15,16]. In complex civilian environments, these competencies suggest that nursing leadership not only plays a supportive role but also constitutes a strategic factor influencing care quality and team resilience [17].

The international literature further highlights that effective leadership is associated with improved nurse satisfaction and retention as well as key indicators such as reduced errors, enhanced patient safety, and greater adherence to evidence-based guidelines [17,18,19]. Taken together, these observations emphasize that the structuring of favorable practice environments by leaders and policy-makers can be considered a measurable determinant of the NPE across both military and civilian settings [20,21].

## 3. Resilience in Crisis Contexts

According to the insights produced in the study by Inácio et al. [1], the military context is characterized by deployments, operational missions, and high-risk situations—elements that increase occupational stress and require resilient NPEs [22]. Studies show that organizational support, role clarity, and interprofessional collaboration reduce stress and improve the psychological well-being of healthcare workers [23,24]. These factors become even more critical in crisis contexts or health emergencies, where operational pressure can compromise safety, quality of care, and staff motivation [25]. Similarly, studies in which civilian nurses have operated in war settings such as Ukraine and Gaza highlight how leadership, structured communication, training, and emotional support are essential to maintaining high performance and team cohesion [9,26,27]. International experience confirms that resilience is not merely an individual quality but the result of a favorable NPE, characterized by adequate staffing, resources, training, and multidimensional/multidisciplinary support [28]. This parallel between military and civilian contexts reinforces the idea that strategies of resilience, leadership, and organizational support can be transferred and structured across different care and responsibility settings, enabling nurses to work effectively even in complex global scenarios such as humanitarian crises, natural disasters, or pandemics [29,30]. “Organizational resilience” therefore emerges as a critical component of the NPE, influencing both the sustainability of the nursing workforce and the quality of care within contemporary care management frameworks. These associations are further supported by Inácio et al. [1] in the military context [31,32].

## 4. Practical Implications and Future Directions

Evidence from both military [1] and civilian contexts highlights strategies for strengthening the NPE. Key approaches include leveraging

- Adaptive leadership and continuous training to develop flexible relational and intercultural skills for diverse teams [33];
- Interprofessional collaboration and participatory decision-making, such as shared governance, structured briefings, and post-crisis debriefings, to enhance engagement, retention, and patient safety [34];
- Adequate staffing and resources to support quality of care and professional satisfaction, particularly in high-demand contexts [35];
- Professional development and career pathways, including continuous education and mentoring, to sustain the NPE and motivate personnel [36,37,38,39];
- Global applicability, allowing these strategies to be adapted to international crisis scenarios where nurses play a central role in public-health protection [40,41,42,43].

## 5. Conclusions

The parallel between military and civilian contexts aims to draw the attention of policymakers and service organizers to the fact that a favorable NPE is crucial for nurse well-being, retention, and patient safety. Nurses could remain at the center of care quality, acting as mediators between organizations, relational processes, and clinical outcomes. The potential parallels between emerging military and civilian evidence may facilitate the development of sustainable and globally relevant strategies, further strengthening the role of the nurse as a possible key figure in care management within global crisis contexts.
